# Machine Learning Supports Long Noncoding RNAs as Expression Markers for Endometrial Carcinoma

**DOI:** 10.1155/2020/3968279

**Published:** 2020-01-10

**Authors:** Ana Carolina Mello, Martiela Freitas, Laura Coutinho, Tiago Falcon, Ursula Matte

**Affiliations:** ^1^Bioinformatics Core, Experimental Research Center, Hospital de Clínicas de Porto Alegre, Porto Alegre 90035-903, Brazil; ^2^Gene Therapy Center, Experimental Research Center, Hospital de Clínicas de Porto Alegre, Porto Alegre 90035-903, Brazil; ^3^Post-Graduation Program on Genetics and Molecular Biology, Universidade Federal do Rio Grande do Sul, Porto Alegre 91501-970, Brazil; ^4^Undergraduation Program on Biotechnology/Bioinformatics, Universidade Federal do Rio Grande do Sul, Porto Alegre 91501-970, Brazil; ^5^Department of Genetics, Universidade Federal do Rio Grande do Sul, Porto Alegre 91501-970, Brazil

## Abstract

Uterine corpus endometrial carcinoma (UCEC) is the second most common type of gynecological tumor. Several research studies have recently shown the potential of different ncRNAs as biomarkers for prognostics and diagnosis in different types of cancers, including UCEC. Thus, we hypothesized that long noncoding RNAs (lncRNAs) could serve as efficient factors to discriminate solid primary (TP) and normal adjacent (NT) tissues in UCEC with high accuracy. We performed an in silico differential expression analysis comparing TP and NT from a set of samples downloaded from the Cancer Genome Atlas (TCGA) database, targeting highly differentially expressed lncRNAs that could potentially serve as gene expression markers. All analyses were performed in R software. The receiver operator characteristics (ROC) analyses and both supervised and unsupervised machine learning indicated a set of 14 lncRNAs that may serve as biomarkers for UCEC. Functions and putative pathways were assessed through a coexpression network and target enrichment analysis.

## 1. Introduction

Uterine corpus endometrial carcinoma (UCEC) is the second most common type of gynecological tumor, in either developed or underdeveloped countries [1]. According to the epidemiologic data from the International Agency for Research on Cancer (IARC) of the World Health Organization (WHO), UCEC comprises 4.8% of cancer incidence worldwide and 2.1% of cancer-related mortality rate [2]. This cancer originates at glandular epithelial cells of the endometrium, which is the mucous layer of the inner uterus [3] and is specified according to clinical and endocrine characteristics by the International Federation of Gynecology and Obstetrics (FIGO): type I carcinomas are estrogen dependent and associated with endometrial hyperplasia, whereas type II carcinomas are estrogen independent and associated with endometrial atrophy [3].

Obesity, aging, early menarche, late menopause, nulliparity, breast cancer, and diabetes mellitus history are some of the endogenous risk factors for developing the disease [4]. Other risk factors include dietetic factors, tamoxifen (https://pubchem.ncbi.nlm.nih.gov/compound/2733526) therapy [5], radiotherapy, and high levels of estrogen [6]. The standard treatment is surgery to remove fallopian tubes and the ovary, which is effective at most cases when treating stage I disease [7]. For advanced stages, surgery followed by treatments such as radiation therapy, chemotherapy, or a combination of both is the best treatment.

Several researches have recently shown the potential of different noncoding RNAs (ncRNAs) as biomarkers for prognostics and diagnosis in different types of cancers [3, 8–10], including UCEC [11, 12] although, currently, no biomarker is consistently used for those purposes in UCEC [13]. Long noncoding RNAs (lncRNAs) are a class of ncRNAs that are at least 200 base pairs long and have multiple functions, albeit these cannot be inferred by their sequences. Most of the lncRNAs are long intergenic (lincRNAs) [14], which are sequences that do not overlap messenger RNAs (mRNAs) [15]. Another class of lncRNAs are antisense RNAs (asRNAs), which are transcribed from the opposite strand of the sense transcripts of either protein-coding or non-protein-coding genes [16]. Some have been associated with chromatin-modifying complexes that confer either repressive or activating modifications [17, 18]; however, the specific functions of the majority of lncRNAs are still unknown.

In UCEC, the lncRNA *HOTAIR* was found to be overexpressed [19] and apparently contributes to the cisplatin-induced resistance by inhibiting autophagy [20]. The silencing of in vivo expression of *HOTAIR* suppressed significantly the endometrial tumorigenesis, leading to smaller tumors [21, 22]. Another lncRNA, *MALAT1*, is known to be overexpressed during endometrial hyperplasia and also during early carcinoma stages; however, its expression is significantly lower in advanced stages, as well as during metastasis [23].

Given their role in modulating UCEC progression, we hypothesized that lncRNAs could efficiently discriminate solid primary tumor (TP) and adjacent normal tissue (NT) with high level of confidence. To test our hypothesis, we performed an in silico differential expression analysis comparing TP and NT from a set of patients, targeting highly differentially expressed lncRNAs that could potentially serve as biomarkers for UCEC. These potential biomarkers were tested using both supervised and unsupervised machine learning. Also, their relationship with other genes was verified by gene coexpression networks and enrichment analysis. From our knowledge, previous works considered only the mRNAs [24, 25], focused in drivers of many cancers (not exclusive) [24], subtypes [26], or other types of uterine carcinoma (nonendometrial) [27]. Similar approaches, based on support vector machine (SVM), have been recently applied to detect biomarkers and key network elements in other tumors [28–31].

## 2. Results and Discussion

From the 46 selected samples (TP and NT from 23 patients) of the total RNA data, we obtained a total of 8,700 transcripts, after normalization and filtering, that went through the differential expression analysis. In order to assess the relationships with other regulatory RNAs and pathways, we also analyzed miRNA data, from which we selected 42 samples (TP and NT from 21 patients, all featured in the total RNA data as well), and 1,881 miRNAs were attained.

**Figure 1:**
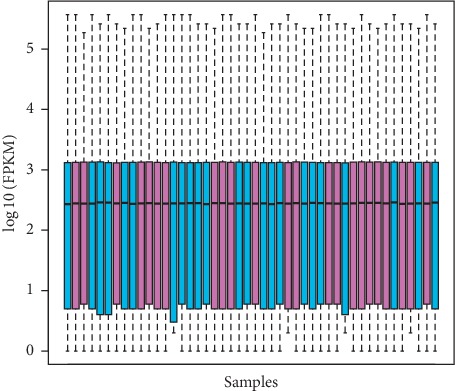
Boxplot after normalization of total RNA samples (training set data). Primary tumor samples (TP) (sky blue); adjacent tumor samples (TP) (purple). Black line inside the boxes indicates the median position. Dotted lines indicate the expression deviations.

**Figure 2:**
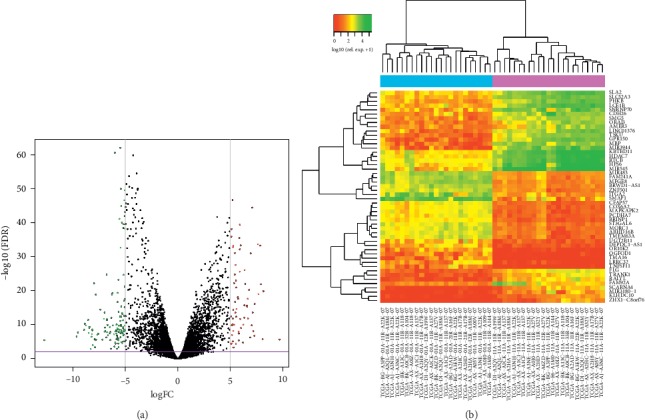
Differentially expressed genes highlighted. (a) Volcano plot of the differential expression analysis of total RNA in primary tumor (TP) compared with adjacent tissue (NT). TP upregulated transcripts (67 genes) with log fold change (logFC) > 5 (red). TP downregulated transcripts with logFC < −5 (124 genes) (green). Horizontal purple line indicates the −log10 (FDR) = 2 (FDR = 0.01) cutoff. Grey vertical lines indicate logFC = −5 and logFC = 5 cutoffs. (b) Heat map of the expression of the 50 most differentially expressed transcripts. Dendrogram: the clustering of TP samples (sky blue); the clustering of NT samples (purple). Top left: colorkey for the heat expression quantification.

The total RNA normalization and filtering of the data guaranteed that the outliers had been removed, and the samples were well normalized (Figure 1), meaning a reduction in the expression deviation and the obtention of an acceptable false positive rate [32, 33]. Both steps indicate that all the differences further found on the expression of the transcripts in TP when compared to NT were most likely caused by the different environments. Considering a |logFC| ≥ 5 and a FDR <0.01 cutoff, a total of 191 transcripts were differentially expressed (Figure 2). Additionally, 67 of them were upregulated in tumor. We chose high values of fold change to ensure that we selected genes with high potential to discriminate the groups and serve as biomarkers.

**Figure 3:**
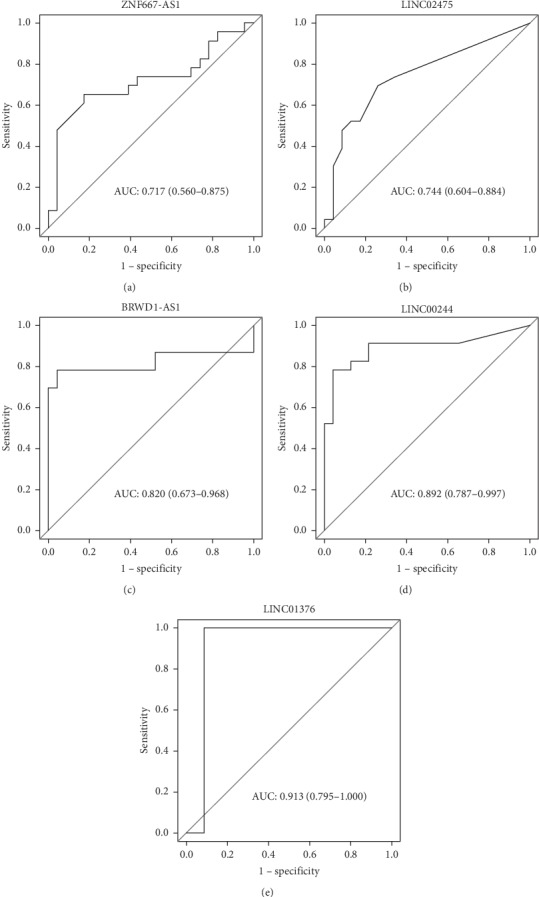
ROC curve of the lncRNAs that obtained AUC greater than 7; sensitivity (true positive rate) (*y* axis); 1-specificity (*x* axis) (false negative rate).

Seventeen lncRNAs were significantly differentially expressed and were chosen as the main candidates to correctly distinguish TP from NT samples (Table 1). Sixteen of them were downregulated in tumor, while only one of them, *LINC01376*, was upregulated, indicating that its expression is essential for maintaining the tumor environment. In order to predict whether the candidates were good for classification models, we plotted AUC-ROC curves for each of them. Five candidates had an AUC greater than 0.7, thus demonstrating high confidence results: *LINC01376*, *BRWD1-AS1*, *LINC00244*, *LINC02475*, and *ZNF667-AS1* (Figure 3).

Three of the top five candidates are lincRNAs. The full range of biological function of lincRNAs remains to be deeply explored [34]. Dysregulation of the expression of lincRNAs may be pervasive in human cancers and drives cancer development and progression [35]. Previous researches demonstrated that there are SNPs on *LINC01376* associated with breast cancer [36, 37]. Notably, it was the only lncRNA upregulated in our analysis with a logFC of 5.88, and it scored the best AUC (0.913) among all candidates.

LncRNAs may be a product of a partially or fully complementary region of a protein-coding gene and may act as a *cis* regulatory lncRNA, in accordance with the sequence complementarity (antisense lncRNA), or may be a lincRNA, acting as a *trans* regulatory element. Also, lncRNAs can connect to DNA, regulating the transcription process, or bind to proteins affecting their stability ([38]). Antisense lncRNAs are known to regulate the transcription output RNA in mammals, affecting the mRNA expression [39, 40] and stabilizing the mRNA [40]. Some antisense lncRNAs may act on their neighboring coding genes, reducing the complementary mRNA expression [41, 42], while others clearly upregulate the expression of the corresponding mRNA and protein [43].

Members of the *BRWD1* gene family are involved in cellular processes such as cell cycle progression, signal transduction, gene regulation, and apoptosis [44]. *BRWD1-AS1* is downregulated in UCEC, with a logFC of approximately −5.05, and scored the third highest AUC (0.820). It is possible that *BRWD1-AS1* could be essential for the normal regulation of the corresponding mRNA, thus its downregulation may lead to cell survival considering that *BRWD1* has an important role in apoptosis. It is important noticing, however, that *BRWD1* was not differentially expressed in our analysis. Another lncRNA-AS that stood out in our analysis was *ZNF667-AS1*, with a logFC of approximately −5.16 and an AUC of 0.717. Other studies have demonstrated that *ZNF667-AS1* is commonly downregulated in several cancer types, including UCEC [36, 37]. Vrba and colleagues [45] showed that it is expressed in all normal finite lifespan human cells examined to date and is downregulated or lost in immortalized human mammary epithelial cells. Additionally, they demonstrated that its downregulation is due to DNA hypermethylation [45]. In cervical cancer, *ZNF667-AS1* inhibited the proliferation of cancer cells and its downregulation was negatively correlated with the overall survival of patients, tumor size, and FIGO stage [46].

**Figure 4:**
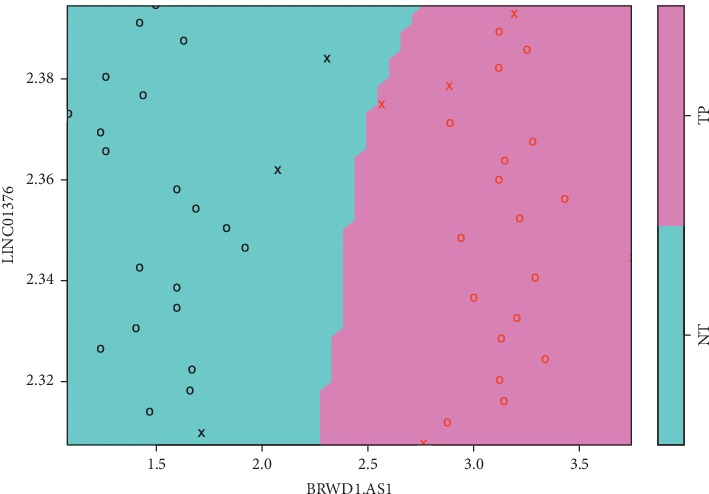
Scatter plot of the fitted support vector machine model based on the expression of the top 2 lncRNAs. Classes: primary tumor (TP) and adjacent tissue (NT). Support vectors instances: x. Expression values are in log10 scale.

**Figure 5:**
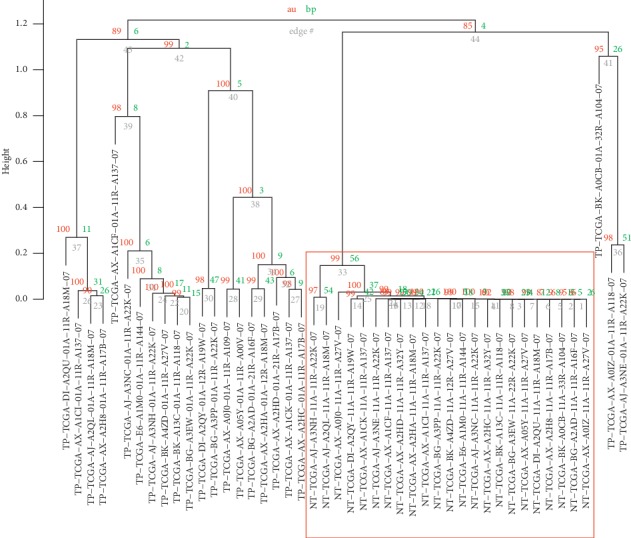
Hierarchical clustering analysis using the train set (23 patients with TP and NT samples for each) based on the expression of the 14 lncRNAs with |log fold change| > 5. Before each sample ID, there is the indication if the samples belong to the TP or NT group. TP: primary solid tumor. NT: adjacent normal tissue. Red values (au) represent the group support. Green values (bp) represent the bootstrap support. Grey values (edge) represent the limit of the branches. Red square highlights the clustering of NT samples.

To evaluate if our combined set of differentially expressed lncRNAs is able to correctly separate TP from NT samples, we performed the SVM approach. From the seventeen lncRNAs, three of them (*PLCG1-AS1*, *LINC01411*, and *LINC02249*) presented elevated expression variation and low AUC and could not be considered good models. Thus, 14 remained during the SVM analysis. The results for all tested sets are shown in Table 2. The first set contained all 14 selected lncRNAs and obtained an accuracy of 0.9583, indicating that this set is an efficient classification model. The second set contained 5 lncRNAs that had an AUC greater than 0.7 and attained an accuracy of 0.9167. The third and final sets contained the top 2 lncRNAs that attained the best overall AUC, obtaining an accuracy of 0.9167 (Figure 4). Thus, even a set composed only by the two top AUC representatives is good enough to discriminate the samples with an accuracy of 91.67%, identical to the accuracy of the top 5 set. The unsupervised hierarchical cluster (Figure 5) demonstrated that all NT samples clustered together with high group support. TP samples were more dispersed and some of them were closer to NTs than to other TPs. This might be explained due to the higher expression variability in TP samples usually observed in many cancer types, such as melanoma [47], breast cancer [48], lung cancer [49], and hepatocellular carcinoma [10].

**Figure 6:**
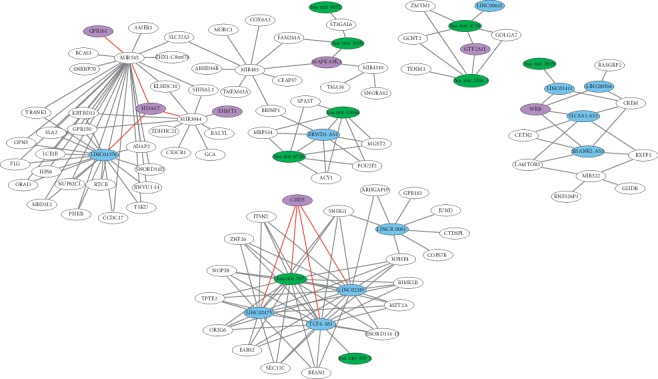
Representative gene coexpression networks. Blue circles are the lncRNAs; green circles are the miRNAs; purple circles are the highlighted genes; white circles are the genes. Each edge represents an *r* > 0.7 or *r* < −0.7 and *p* value < 0.05. Red edges highlight the discussed pathways.

In order to look for pathways and biological functions of the lncRNAs from our set, we performed a correlation analysis among the expression data of all differentially expressed transcripts (including miRNAs). A gene coexpression network was plotted from the result of the correlation analysis (Figure 6) and was assessed alongside enrichment analyses. ClueGO (v. 2.3.3) [50] indicated that CHD5 and WRB (downregulated) and EHMT1 (upregulated) were involved in the histone H3K27 methylation. Trimethylation of H3K27 has been associated with transcriptional inhibition of genes in endometrial cancer [51]. In addition, EZH2, which is a methyl-transferase for H3K27, is upregulated in many tumors [52, 53], consequently causing low expression levels of the genes regulated in this part of the genome. In this study, EZH2 is slightly upregulated in UCEC, although the difference was not statistically significant, considering a FDR <0.01. Previous studies have demonstrated that downregulation of H3K27 methylation process in tumor may contribute to its progression by enhancing the expression of oncogenes [54, 55].

Many studies first identified CHD5 as a tumor suppressor gene in neuroblastomas [56–60]. Subsequent research showed increasing evidence that it functions as tumor suppressor in several other types of cancer, including ovarian [61], breast [62], lung [63], and colorectal cancer [64]. Thus, CHD5, commonly found downregulated in cancer due to deletion of region 1p36, where it is located, may act as a master regulator, controlling the key processes for the suppression of a variety of tumors [65]. Clonal alteration was found at the region 1p36 in endometrial cancer [66], which might explain why CHD5 is downregulated with a logFC of -5.40 in our analysis. Furthermore, in our coexpression network, CHD5 is positively and strongly linked to three lncRNAs of our set: *TCF4-AS1*, *LINC02249*, and *LINC02475*, which may indicate that they possibly play a role on the expression of CHD5. To our knowledge, all three lncRNAs have not been biologically associated with cancer to date, nor to any other disease. CHD5 was also positively correlated to hsa-mir-767, which is downregulated in UCEC. Downregulation of this miRNA has also been detected in lung adenocarcinoma cells [67], whereas upregulation was detected in human melanoma [68]. No miRNA prediction binding site tool (see Section 4) identified a statistically supported connection between hsa-mir-767 and CHD5.

Gene ontology enrichment analysis performed on pathfindR (v. 1.2.1) [69] showed that *GPR161* (upregulated) was associated with the negative regulation of the hedgehog signaling pathway. However, previous studies revealed that abnormal activation of this pathway is related to cell proliferation in endometrial cancer [70, 71]. According to the Human Protein Atlas [72], high expression of *GPR161* is associated with low survival probability in UCEC (66% probability of 5-year survival in GPR161 high and 79% in GPR161 low, *p*=0.0016) but it is not prognostic for UCEC. Additionally, *GPR161* has been found to be upregulated in breast cancer as well, acting as a promoter of cell proliferation and invasion [73]. The coexpression network reveals that this gene is positively correlated with MIR545, a pre-mir, which has been linked to cell proliferation in colorectal cancer [74] and hepatocellular carcinoma [75]. We also found that MIR545 is upregulated in endometrial cancer with a 7.8 logFC. Those results indicate that *GPR161* and MIR545 are probably promoting cell proliferation on UCEC as well, but other elements may also be involved. Again, no miRNA prediction binding site tool (see Section 4) identified a statistically supported connection between MIR545 and *GPR161*.

KEGG analysis also performed on pathfindR (v. 1.2.1) [69] indicated that the upregulated genes *HDAC7*, *GTF2A1L*, and *MAPKAPK2* were involved in the viral carcinogenesis pathway. Previous studies have demonstrated that inhibition of histone deacetylases, such as *HDAC7*, induces apoptosis, cell cycle arrest, and growth inhibition in endometrial cancer cells [76–79]. Moreover, *LINC01376*, the only lncRNA that is upregulated in UCEC from our set, positively and strongly correlates with *HDAC7* (*r* = 0.91), which reinforces *LINC01376* as a marker. Again, based on the Human Protein Atlas [72], high expression of *HDAC7* is associated with low survival probability in UCEC (*p*=0.045), but it is not considered a prognostic for UCEC. Based on KEGG (https://www.genome.jp/kegg/), *MAPKAPK2* also participates in cellular senescence.

In order to look for more putative pathways and biological functions of the lncRNAs from our set, we performed an *in silico* prediction of lncRNA function. As stated before, characterization of most lncRNAs remains to be done; therefore, we were able to predict the functions of seven lncRNAs from our set: *BRWD1-AS1*, *LINC00504*, *SHANK2-AS1*, *MACC1-AS1*, *WRD86-AS1*, *LINC00632*, and *ZNF667-AS1*. The first four were strongly associated with male reproductive system pathways, such as spermatogenesis, male gamete generation, and sperm motility. The alteration of this pathway is not a novelty in endometrial cancer [80, 81]. *LINC00632* and *ZNF667-AS1* were potently related with neural functions such as neuron projection, synapse organization, and neurotransmitter secretion and transport, whereas *WRD86-AS1* was linked to the spliceosome complex and RNA splicing, as well as to breast, thyroid, and colorectal cancers.

Even though there have been lncRNAs reported as oncogenic drivers [82], survival analysis performed for each lncRNA from our set did not associate their expression profile to a specific prognostic; consequently, none of them can be reckoned as drivers. However, these lncRNAs can still act as biomarkers, once we demonstrated that they effectively distinguish TP from NT.

## 3. Conclusions

In summary, our data suggest a set of 14 lncRNAs as highly effective biomarkers of UCEC, albeit none can be reckoned as drivers and coexpression associated genes are not prognostic. Moreover, this set can be reduced to the two top lncRNAs (*LINC01376* and *BRWD1-AS1*) with a minimal reduction in accuracy and specificity. This data must be validated in clinical samples, but it is predicted to contribute in the diagnosing UCEC. While there have been similar works on lncRNAs [83] that paved the way for our study, we focused on looking for prognostics and diagnostics biomarkers for UCEC, considering only highly expressed lncRNAs and honing on strong statistical analyses to support our findings. In addition, the elucidation of how these lncRNAs play a part in the establishment and progression of UCEC may contribute to new diagnostic options in the future.

## 4. Materials and Methods

All data analyzed in this study are available at the Cancer Genome Atlas (TCGA), and all analyses were performed using the R software (v. 3.4.0) (https://www.R-project.org/). Data download, preprocessing, and differential expression analysis were performed using TCGABiolinks package (v 2.7.1) [84], available at Bioconductor digital repository (https://www.bioconductor.org/). Total RNA and miRNA data must be separately downloaded from TCGA. For the total RNA data, 587 samples were downloaded: 35 NTs and 551 TPs. We analyzed an initial set of 23 patients that had both NT and TP samples, thus a total of 46 samples. The results were then visualized on volcano plots, and the 50 most differentially expressed transcripts (top 50) were featured on a heatmap, plotted using the package gplots (v. 3.0.1). For the miRNA data, a total of 579 samples were downloaded, 33 NTs and 545 TPs. We analyzed a set of 21 patients (all included in the total RNA analysis) that had both TP and NT samples, thus a total of 42 samples.

For the putative lncRNA biomarkers, we first performed a supervised prediction model using the area under the curve (AUC) of the receiver operating characteristic (ROC) with the pROC package (v. 1.13.0) [85], then a supervised learning model using support vector machines (SVM), with the caret package (v. 6.0–81). Next, we performed an unsupervised model using hierarchical cluster analysis with 1000 bootstrap replications. Clusters with unbiased grouping support *p* values (au) of approximately 95% were considered as statistically significant groups. Hierarchical clusters were plotted using the pvclust package (v. 2.0–0) [86]. We also performed a Kaplan–Meier survival analysis considering the expression of the lncRNAs only on TP samples for the purpose of appraising whether they could be considered drivers for UCEC. At last, in order to look for pathways and biological functions of the lncRNAs of our set, we set up a coexpression network that was assessed alongside enrichment analyses. We also used FuncPred [87], an online tool that performs in silico prediction of lncRNA function by using tissue specific and evolutionary conserved expression.

### 4.1. Download and Data Preprocessing

The UCEC harmonized total RNA expression data (hg38) and miRNA expression data downloaded from TCGA were obtained through RNA-seq using Illumina HiSeq platform. Total RNA and miRNA are separately download due to differences in the data composition. First, we set the query for the download of the total RNA data, using the function *GDCquery* with the following options: *project* *=* *“TCGA-UCEC,” data.category* *=* *“Transcriptome Profiling,” data.type* *=* *“Gene Expression Quantification,” workflow.type* *=* *“HTSeq—Counts”* and *legacy* *=* *FALSE*. The query for the microRNA data uses the following options: *project* *=* *“TCGA-UCEC,” data.category* *=* *“Transcriptome Profiling,” data.type* *=* *“miRNA Expression Quantification”* and *legacy* *=* *FALSE*. Then, we downloaded both data using the function *GDCdownload* with the previously set queries and the option *method* *=* *“api*.*”* The function *GDCprepare* was then used to transform the downloaded total RNA data into summarized experiment data, making it suitable for the analyses.

The data normalization was performed first by GC content, using the function *TCGAanalyze_Normalization* with the options *geneInfo* *=* *geneInfoHT* and *method* *=* *“gcContent,”* and then by gene length, changing the *method* option to *“geneLength.”* Then, a quantile filter was applied using the function *TCGAanalyze_Filtering* with the option *method* *=* *“quantile”* and *qnt.cut* *=* *0.25*. Finally, the Spearman correlation among samples was checked using the R function *cor* and both NT and TP sample groups were separated with the function *TCGAquery_SampleTypes*.

### 4.2. Differential Expression and Survival Analysis

We considered as differentially expressed those genes with a false discovery rate (FDR) < 0.01 and log fold change (logFC) ≥ |±5|, using the function *TCGAanalyze_DEA* with the options *fdr.cut* *=* *0.01*, *logFC.cut* *=* *5*, *and method* *=* *“glmLRT.”* This high cutoff was chosen in sense to detect the genes with the highest potential for group differentiation.

Considering the expression on TP samples of all differentially expressed lncRNAs, we performed a Kaplan–Meier survival analysis with the function *TCGA_SurvivalKM and the following options: Survresult* *=* *TRUE*, *p.cut* *=* *0.5*, *ThreshTop* *=* *0.67*, and *ThreshDown* *=* *0.33*. Survival curves for each lncRNA were generated and analyzed separately.

### 4.3. ROC Analysis

For the ROC analysis, we focused in the differentially expressed lncRNAs. The individualized results were visualized on a AUC-ROC curve plot, which is a graph showing the performance of a classification model at all classification thresholds. The evaluation metric used was the AUC, which represents the degree or measure of separability, that is, how much the model is capable of distinguishing between two groups [88].

### 4.4. Support Vector Machine (SVM) Analysis

For the SVM, we first set a randomly chosen seed of 3033, making our work replicable, with the function *set.seed (3033)*. Then, we used the function *trainControl()* that controls the computational nuances of the *train()* method. At this point, we applied a 1000 bootstrap replication, with the option *method* *=* *“boot”* and *number* *=* *1000*.

Next, we set our train and prediction data. The train data contained the selected differentially expressed lncRNAs from the 23 initial patients. Here, three lncRNAs were cut out of the analysis for presenting elevated expression variation. Our prediction model data contained the same lncRNAs, however, from 24 samples, different from the 46 samples we have been working on: 12 TPs and the remaining 12 NTs from TCGA database that did not have TP/NT correspondence in the same patient. The expression data from the 24 new patients were analyzed apart from the set of 23 patients. To train our data, we tested both land radial kernel methods, using the function *train()* with the previously set *trainControl* as a parameter, along with the options *preProcess* *=* *c (“center,” “scale”)*, *tuneLength* *=* *10*, and *method* *=* *“svmLinear”* or *method* *=* *“svmRadial”*. Next, we tested our classifier at specific cost (C) values for linear method, using the function *expand.grid* with cost values ranging from 0 to 5 and adding the *tuneGrid* parameter with the set grid to the *train()* function.

This process was repeated for three different sets of lncRNAs. The first set contained all selected lncRNAs, and the best performance was obtained using linear kernel method, with cost = 0.25. The second set had the ones that attained an AUC greater than 0.7. The best performance was obtained using the linear kernel method, with cost = 1. The third set had the two lncRNAs that attained the best AUC. The best performance was obtained using a radial basis function kernel method, with standard values of sigma = 0.07 and cost = 5.

### 4.5. Coexpression Network and Enrichment Analysis

A Spearman correlation analysis among the expression data of all differentially expressed transcripts and miRNAs was performed in order to plot a gene coexpression network. The cutoff for the statistically significant correlations was *r* > 0.7 and *r* < −0.7 and *p* value <0.05. The network was visualized using Cytoscape (v. 3.5.1) [89]. For better clearance of the network, only the following correlations of interest were plotted: lncRNA-miRNA, lncRNA-mRNA, and miRNA-mRNA. The coexpression network was analyzed alongside enrichment analyses performed on ClueGo (v. 2.3.3) [50] and pathfindR (v. 1.2.1) [69].

### 4.6. miRNA Interaction Prediction

The miRNAs and their putative targets were used to predict their interaction using the online software TargetScan (release 7.1) [90] and miRDB [91].

## Figures and Tables

**Table 1:** Long noncoding RNAs that discriminate TP from NT samples.

lncRNA	logFC	FDR	AUC
*LINC01376*	5.88492063377296	2.37514982980919*e*^−26^	0.913
*BRWD1-AS1*	−5.05883480566089	2.3408685424323*e*^−50^	0.820
*LINC00244*	−5.11057542483241	2.25688066033936*e*^−13^	0.892
*LINC02475*	−5.15921206745447	8.93670161058092*e*^−06^	0.744
*ZNF667-AS1*	−5.16386849714406	8.14134337771604*e*^−08^	0.717
*DEPDC1-AS1*	−5.17825782522368	2.06667461632931*e*^−25^	0.633
*LINCR-0001*	−5.20814071478136	4.04097876674846*e*^−10^	0.601
*LINC00504*	−5.29670533561579	2.78034526756707*e*^−08^	0.535
*LINC00632*	−5.38175032295688	4.77496645881152*e*^−16^	0.659
*MACC1-AS1*	−5.38578795348022	2.25613841195626*e*^−10^	0.694
*TCF4-AS1*	−5.64269979170812	0.000474000179607125	0.516
*SHANK2-AS1*	−5.92292108357159	4.02699887083864*e*^−05^	0.624
*WDR86-AS1*	−6.32585914185104	9.39257125151877*e*^−13^	0.643
*LINC01411*	−7.78029289441235	2.81489971171908*e*^−06^	0.471
*PLCG1-AS1*	−7.93247628000136	1.81453269242651*e*^−06^	0.674
*SLC8A1-AS1*	−8.15747923329883	4.51757852734348*e*^−09^	0.511
*LINC02249*	−9.64327159184569	2.76479236381279*e*^−07^	0.578

**Table 2:** SVM analysis for the three tested sets of lncRNAS.

Set^*∗*^	Accuracy	CI (95%)	*p* value	Kappa	Sensitivity	Specificity
First set	0.9583	0.7888, 0.9989	1.49*e* − 06	0.9167	0.9167	1.0000
Second set	0.9167	0.6262, 0.9526	1.794*e* − 05	0.8333	0.9167	0.9167
Third set	0.9167	0.73, 0.9897	1.794*e* − 05	0.8333	0.9167	0.9167

^*∗*^The first set is comprised of 14 lncRNAs; the second set is composed of the five lncRNAs with AUC > 0.7; the third set is composed by the AUC's top two lncRNAs, as shown in Table 1.

## Data Availability

All data used in this work are publicly available at the Cancer Genome Atlas (TCGA) database (https://cancergenome.nih.gov/).
